# Effects of Scheduled Exercise on Cancer-Related Fatigue in Women with Early Breast Cancer

**DOI:** 10.1155/2014/271828

**Published:** 2014-01-19

**Authors:** Anne Marie Lunde Husebø, Sindre Mikal Dyrstad, Ingvil Mjaaland, Jon Arne Søreide, Edvin Bru

**Affiliations:** ^1^Department of Health Studies, University of Stavanger, 4036 Stavanger, Norway; ^2^Department of Education and Sports Science, University of Stavanger, 4036 Stavanger, Norway; ^3^Department of Oncology, Stavanger University Hospital, 4068 Stavanger, Norway; ^4^Department of Gastroenterological Surgery, Stavanger University Hospital, 4068 Stavanger, Norway; ^5^University of Bergen, 5021 Bergen, Norway; ^6^Norwegian Centre for Learning Environment and Behavioural Research in Education, University of Stavanger, 4036 Stavanger, Norway

## Abstract

While physical activity during cancer treatment is found beneficial for breast cancer patients, evidence indicates ambiguous findings concerning effects of scheduled exercise programs on treatment-related symptoms. This study investigated effects of a scheduled home-based exercise intervention in breast cancer patients during adjuvant chemotherapy, on cancer-related fatigue, physical fitness, and activity level. Sixty-seven women were randomized to an exercise intervention group (*n* = 33, performed strength training 3x/week and 30 minutes brisk walking/day) and a control group (*n* = 34, performed their regular physical activity level). Data collection was performed at baseline, at completion of chemotherapy (Post_1_), and 6-month postchemotherapy (Post_2_). Exercise levels were slightly higher in the scheduled exercise group than in the control group. In both groups, cancer-related fatigue increased at Post_1_ but returned to baseline at Post_2_. Physical fitness and activity levels decreased at Post_1_ but were significantly improved at Post_2_. Significant differences between intervention and control groups were not found. The findings suggest that generally recommended physical activity levels are enough to relief cancer-related fatigue and restore physical capacity in breast cancer patients during adjuvant chemotherapy, although one cannot rule out that results reflect diminishing treatment side effects over time.

## 1. Introduction

Physical activity guidelines recommend healthy individuals to perform 150 minutes·wk_1_ of at least moderate-intensity physical activity (MVPA), in order to obtain health-promoting effects [1]. Breast cancer survivors are advised to avoid inactivity and to follow the same age-appropriate guidelines as healthy individuals [2]. The effects of exercise on treatment-related issues in cancer patients may fade rapidly if not maintained or if the exercise is not sufficient enough [3]. Thus, evaluating the patients' adherence to physical activity guidelines is important. However, as shown by others, many cancer patients reduce their physical activity after the diagnosis, or they exercise less than recommended [4]. A study of 1,696 breast cancer survivors found a decrease in metabolic equivalent of task (MET) hours per week by a mean of −9.40 (27.94), corresponding to 30 minutes of MVPA 4.7 days per week [5].

It has been reported that receiving chemotherapy is one of the strongest independent predictors for reduced physical activity level [5]. Many patients are offered a combination of both hormonal and cytotoxic treatments, with or without trastuzumab. In Norway, adjuvant chemotherapy usually comprises an 18-week treatment with anthracycline-based polychemotherapy (fluorouracil, epirubicin, and cyclophosphamide; FEC). Some patients are treated for 3 months with this combination, followed by a 3-month period of taxane monotherapy [6]. Due to treatment effects on nonneoplastic cells, severe physical, emotional, and cognitive treatment-related symptoms may appear during or shortly after the delivery of chemotherapy [7, 8]. Cancer-related fatigue is considered the most prevalent and distressing symptom in relation to cancer treatment and has been defined as “an overwhelming sustained sense of exhaustion and decreased capacity for physical and mental work at usual level” [9]. It can occur from the time of diagnosis, through treatment, and is present in about 26–28% of breast cancer patients following treatment [7]. Studies report that fatigue shows a high and fluctuating prevalence similar to a roller-coaster pattern during adjuvant chemotherapy [10] and may be present for as long as 5 years following treatment with no improvement during the first two years [7, 10]. Fatigue as a side effect of the cancer treatment has a negative effect on physical fitness and physical activity levels [7, 11].

Several physical activity interventions aiming at improving health outcomes during cancer treatment have been tested, as demonstrated by the numerous, recent literature reviews [12–18]. Of seven reviews published between 2007 and 2012, breast cancer patients were the most studied cancer population, representing from 46–100% of the studied populations. The reviews identified ambiguous findings concerning the effect of scheduled exercise interventions on cancer-related fatigue [14–18]. Considerable heterogeneity between exercise intervention studies, regarding exercise mode, outcome measurements, and disease- and treatment-specific factors, has been highlighted as a possible explanation [15, 18]. Moreover, the reviews give limited information on recommended exercise dose (i.e., frequency, intensity, and duration) and do not include comparison of effects of scheduled exercise interventions as compared to general recommendations on physical activity [2]. This represents a lack of clarity whether explicit exercise interventions will reduce negative treatment side effects and give effects additional to that of simply advising the women to be physically active according to general recommendations.

In this study, effects of a scheduled home-based exercise intervention on cancer-related fatigue, physical fitness, and physical activity level, as compared to being advised to exercise at a regular physical activity level, was investigated among breast cancer patients. The exercise intervention combined resistance and aerobic exercise, to be performed during chemotherapy treatment. Regular physical activity level was defined as physical activity in accordance with general recommendations of 150 minutes/week of MVPA. We hypothesized that the scheduled exercise intervention program would significantly reduce cancer related fatigue and increase physical fitness and physical activity levels compared to general recommendations of physical activity and that these changes would be significantly greater in the intervention group compared to the control group.

## 2. Methods

### 2.1. Study Design and Population

The randomized, controlled trial was conducted in one university hospital in Norway during 2010–2012. Eligible breast cancer patients were between 18 and 70 years of age, surgically treated for early stage breast cancer (mastectomy or lumpectomy), and allocated to adjuvant chemotherapy according to the national treatment guidelines of the Norwegian Breast Cancer Group. The included patients had to be able to read, write, and speak Norwegian, and they were approved for participation in this study by a clinical oncologist. The random assignment of subjects to the intervention group or to the control group was carried out by the use of concealed envelops, drawn by the research assistant prior to the first data collection.

### 2.2. Ethics

The study was conducted in accordance with the Declaration of Helsinki (1964), and approved by the Norwegian Regional Committees for Medical and Health Research Ethics (Reg. No. 2009/2283). All participants gave their volitional, written consent based on both verbal and written information on the characteristics of the intervention program and assessment procedures provided by the clinic staff.

### 2.3. Data Collection Procedures

The study sample completed questionnaires and physical tests after surgery prior to chemotherapy (baseline), 18–24 weeks after baseline and at the end of chemotherapy (Post_1_), and approximately six months after completing the chemotherapy regimen (Post_2_). Demographic characteristics were obtained by a questionnaire (age, body weight, height, ethnicity, marital status, living conditions, education, and employment). Clinical data were retrieved from the hospital records (diagnosis, cancer stage, surgical treatment, lymph node status, hormone receptor status, adjuvant treatment, previous cancer history, and other health conditions). In addition, the questionnaire provided data on cancer related fatigue and physical activity levels. Physical fitness was assessed by a physical test, and data on exercise volume were collected from exercise diaries.

*(1) Schwartz Cancer Fatigue Scale (SCFS-6).* Cancer-related fatigue was measured by a revised version of the SCFS-6, a 6-item scale developed to measure cancer specific fatigue on two dimensions: physical and perceptual and on a 1 “not at all” to 5 “extremely” scale [19]. Sum scores range from 6 to 30 a higher score indicating the subject feeling more fatigued. Content and constructs validity and reliability have been demonstrated, with a Cronbach's alpha for the total scale of 0.90 [19, 20]. In this study SCFS-6 was translated from English to Norwegian by a standard back-translation procedure [21]. Two bilingual persons, both fluent in Norwegian and English, translated and back-translated until agreement was reached. The Norwegian version of SCFS-6 was then content validated by two health professionals and a breast cancer patient. A Cronbach's alpha of 0.83 indicated good internal consistency of the Norwegian version of SCFS-6. 

*(2) International Physical Activity Questionnaire (IPAQ) Short Form.* Physical activity level was assessed by the IPAQ short form. The IPAQ short form was employed as a supplement to exercise diaries, to ensure recording of activity levels between Post_1_ and Post_2_, since the participants did not report in exercise diaries in this time period. In the IPAQ short form the participants were asked to recall their physical activities during the last 7 days [22]. The IPAQ short form gives information on metabolic equivalent of task (MET) hours during moderate and vigorous activity and minutes spent sitting down. IPAQ short form scoring guidelines provide three physical activity levels: *low* (i.e., <600 MET-minutes/week), *moderate* (i.e., ≥600 MET-minutes/week), and *high* (i.e., ≥3000 MET-minutes/week) [23]. Through extensive reliability and validity testing the IPAQ Executive Committee has approved the IPAQ short form in many countries for comparing population estimates for physical activity [22].

*(3) 6-Minute Walk Test (6-MWT).* Physical fitness was assessed by the 6-MWT which measures how far the patient can quickly walk on a flat, hard surface during a 6-minute time period. It assesses the patient's functional capacity on a sub-maximal level and reflects the exercise level for daily physical activities [24]. A healthy individual's 6-MWT range from 400 to 700 meters (m), and an improvement of more than 70 m is considered to be of clinical importance to the patient [25].

*(4) Exercise Diary.* Exercise volume was obtained from exercise diaries, in which the participants in both groups registered their daily exercise activities and leisure time activities (e.g., gardening). The registration started at baseline and lasted until two weeks after the last cycle of chemotherapy. Weekly exercise minutes were calculated for each activity type. For the walking regimen, weekly minutes were calculated on all four intensity levels. Total physical activity intensity categorization was calculated for both groups, and included strength training with rubber bands, walking, and additional strength training and aerobic exercise. Examples of additional strength training were yoga, pilates, and weight lifting, while examples of additional aerobic exercise were spinning, jogging, and swimming.

### 2.4. Exercise Intervention

The intervention consisted of a home-based exercise program that combined strength and aerobic training performed throughout the time period of adjuvant chemotherapy. The strength training prescription included exercises with resistance bands for arms and legs and strength training for the upper body, and the subjects were recommended to perform this training three times per week. The aerobic prescription consisted of a daily 30 minutes of brisk walking, which could be split into periods of 10-minute walks. Patients were instructed to categorize the walking intensity in four different intensity levels (light, moderate, vigorous, and very vigorous) [26]. They were encouraged to obtain at least moderate intensity during walks. The women in the intervention group were supported and encouraged in their exercise by motivational telephone calls from the research team every second week. The telephone calls were also used to monitor adverse events. The women in the control group were encouraged to remain on their regular activity level and received one follow-up call during the intervention time period.

### 2.5. Exercise Adherence

Exercise adherence was defined as the extent to which the women in the intervention group performed the exercise program as prescribed, operationalized as walking at moderate intensity for at least 30 minutes per day (i.e., 210 minutes/week) and performing the strength training program at least three times per week. Data on adherence was obtained from the exercise diaries.

### 2.6. Statistical Analysis

A power analysis was performed to determine the sample size for this study, using the statistical analysis program G*Power [27, 28]. Power analyses indicated a sample size of 38 for medium effect sizes (Cohen's *f* = 0.25) and 58 when expecting small effect sizes (Cohen's *f* = 0.20). A planned sample size was set to 60 participants. Statistical analyses included descriptive analyses, reliability testing, one-way ANOVA, and a mixed design ANOVA conducted by the GLM-procedure in SPSS [29]. The mixed design ANOVA procedure allows a mixture of between-group and repeated measures variables and thereby tests the significance of within- and between-group differences simultaneously. This technique was employed to examine the effectiveness of the exercise program. In addition, Cohen's *d* correcting for dependence between means scores was calculated. Patterns of missing data on the Schwartz Cancer Fatigue Scale-6 (SCFS-6) were registered by each case for all three time points. No case had more than two missing items. Missing items were replaced by a computed mean based on the scores on the remaining items. Missing data on the International Physical Activity Scale (IPAQ) short form were handled by excluding cases missing more than two of the activity intensities. Cases that reported activity on 1-2 of the intensities and those who reported zero activity on all three intensity levels remained in the analysis. Statistical significance was set at *P* < 0.05. A *P* value between 0.05 and 0.1 indicated a tendency. All statistical analyses were performed using PASW Statistics 18 for Windows [29]. Inspections of the distributions of dependent variables revealed that scores for the SCFS-6 and the 6-Minute Walk Test showed approximately normal distributions and were suited for parametric statistics. IPAQ short form deviated somewhat from the normal distribution (skewness 1.73–2.24; kurtosis 2.75–4.85) and follow-up analysis implementing scores transformed by the lg10 algorithm was performed.

## 3. Results

### 3.1. Patients Characteristics

**Figure 1:**
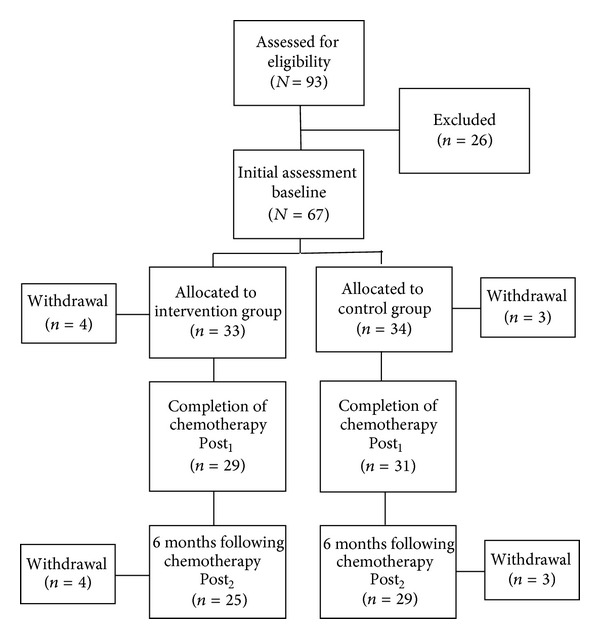
Flow of breast cancer patients through the trial.

The flow of participants through the study is presented in Figure 1. Among 93 consecutive and eligible breast cancer patients, 67 (72%) patients agreed to participate and completed the questionnaires and performed the physical tests at baseline. Seven patients (10.4%) withdrew from the study before the second data collection at Post_1_, leaving 60 patients to complete the data collection. At Post_2_, 52 patients (77.6% of the baseline sample) remained in the study and completed questionnaires and physical tests for the last time, resulting in a total drop-out rate of 22.4%. The representativeness of the follow-up sample was tested by ANOVA and cross-tabulations including chi-square tests. At baseline the mean age of the women in the intervention group was 50.8 and 53.6 in the control group. Most of the women in both groups were of Norwegian origin and living with a partner. 43.3% of them had children living at home, and 49.3% were employed. In the intervention group, 59.3% had a university degree, whilst 35.2% in the control group had a university degree. Most of the women in both groups had undergone breast-conserving surgery and were diagnosed with cancer stage I or II. Half of the women in both groups received a chemotherapy regimen consisting of both FEC (i.e., fluorouracil, epirubicin, and cyclophosphamide) and taxane, and 71.7% of the total sample received radiotherapy, following the chemotherapy treatment. No significant difference between those completing the follow-up test and those that withdrew was found for demographic and clinical variables, fatigue, 6-Minute Walk Test, and total exercise volume for moderate to vigorous physical activity.  Table 1 shows baseline characteristics of the total study sample and of the intervention and control group.

### 3.2. Exercise Volume

Exercise volume recorded in individual exercise diaries showed that the patients exercised for 17 weeks on average (Table 1), with a mean of 168 (SD 100) minutes of moderate to vigorous physical activity (MVPA) per week. The intervention group had a mean exercise volume of 194 (SD 110) minutes of MVPA. While 58% met the general recommendations of 150 minutes/week of MVPA, only 17% adhered to the walking prescription of minimum 210 minutes/week of MVPA. Participants carried out approximately two sessions of resistance band exercises per week, and 15% of the participants in the intervention group achieved the prescribed number of strength training sessions. The control group had a mean exercise volume of 144 (SD 84) MVPA minutes per week, and 39% performed 150 minutes/week of MVPA or more. Data on exercise volume indicates that 48% of participants in both groups exercised according to the general recommended physical activity level or more. However, there was a tendency of a significantly larger mean exercise volume in the intervention group compared to the control group (*P* = 0.051, Cohen's *d* = 0.52).

Adverse events related to the exercise intervention were few. One participant in the intervention group reported knee discomfort and was referred to her primary physician for further evaluation. The patient stayed in the trial and completed the exercise prescription. Another participant in the intervention group experienced syncope during the walking exercise. This was related to a secondary chronic condition, and the patient was advised by her oncologist to withdraw from the trial.

### 3.3. Cancer-Related Fatigue

In general, low mean fatigue scores for both the intervention group and the control group at all three time points (Schwartz Cancer Fatigue Scale-6; range 6–30) were encountered (see Table 2). The results in Table 3 show that the fatigue scores increased significantly from baseline to end of chemotherapy for the whole sample (*P* = 0.003; Cohen's *d* = 0.41). Comparison of mean fatigue scores at baseline and Post_2_ showed a nonsignificant difference (*P* = 0.181; see Table 4), indicating a return to baseline levels of fatigue for the total sample. No significant differences in the trajectory of fatigue between exercise and control groups were found.

### 3.4. Physical Activity Level

Physical activity levels measured by the International Physical Activity Questionnaire-short form showed that the total sample can be classified as exercisers at a moderate level of physical activity (i.e., ≥600 metabolic equivalent of task (MET)-minutes/week) at all three points of measurements (see Table 2). Tables 3 and 4 show that mean levels for MET-minutes/week did not change significantly from baseline to Post_1_ for the total sample but increased significantly from baseline to Post_2_ (*P* = 0.00; Cohen's *d* = 0.62). No significant differences in changes in mean levels of MET-minutes/week between the intervention and control groups were found. Follow-up analysis with lg10 transformed scores yielded results in accordance with those presented in Tables 3 and 4.

### 3.5. Physical Fitness

Mean physical fitness measured by the 6-Minute Walk Test (6-MWT) is presented in Table 2, and results for tests of changes in mean scores between groups and points of measurement are presented in Tables 3 and 4. For the total sample physical fitness decreased marginally from baseline to Post_1_ (*P* = 0.088), whereas the test of baseline-Post_2_ changes showed a significant increase (*P* = 0.009; Cohen's *d* = 0.39). No significant differences in changes for the 6-MWT between the two groups were demonstrated.

## 4. Discussion

The present study examines effects of a scheduled aerobic exercise and strength training intervention on cancer-related fatigue, physical fitness, and physical activity levels during adjuvant breast cancer chemotherapy compared to general recommended physical activity. The results provide information on exercise dose (frequency, intensity, duration, and mode) sufficient to relieve treatment-related symptoms and restore physical capacity in breast cancer patients during adjuvant chemotherapy.

Although there is inconsistent evidence regarding persistence of cancer-related fatigue [11], it has been found to be a long-lasting side effect of cancer treatment [7, 30]. A positive finding from our study was that the mean fatigue levels returned to pretreatment levels 6 months after the end of chemotherapy in both the intervention group and the control group. Especially, considering that nearly 72% of our participants received radiotherapy following the chemotherapy and that radiotherapy is known to cause elevated fatigue levels in breast cancer patients [31], one would expect increased fatigue levels also at the 6-month followup. The fatigue experience followed the same trajectory in both conditions, increasing towards the end of the chemotherapy time period (Post_1_) and returning to initial assessment levels 6 months after the chemotherapy treatment had been completed (Post_2_). The pattern of results for physical fitness and physical activity levels appeared slightly different, showing a significant improvement in walking distance and activity levels 6 months after completing the chemotherapy. This might point towards increased energy levels and can be described as a consequence of relatively high exercise activity during treatment in this sample.

As for cancer-related fatigue, physical fitness and physical activity levels showed the same changes over time in the intervention group and the control group. The recommendations from American College of Sports Medicine state are that it is safe and effective for breast cancer patients to perform moderate to vigorous physical activity (MVPA) 150 minutes/week [1]. In this study self-reported mean physical activity levels in both groups met the generally recommended weekly MVPA, and the study sample exercised according to a moderate activity level as measured in MET-minutes/week. Results could thus reflect that both groups exercised enough to relief negative effects of chemotherapy on the studied outcomes and that the scheduled exercise intervention did not give additional effect.

The findings of this trial should be interpreted with caution due to some limitations. The employed research design does not make it possible to control for changes in the effects of cancer therapy on dependent variables over time. Although elevated levels of cancer-related fatigue and reduced physical fitness and activity levels should be expected also six months after chemotherapy [32], one cannot rule out that diminishing side effects of chemotherapy could explain the results. A possible explanation why participants in the intervention group did not achieve effects beyond the control group at this point of measurement could be the relative small difference in exercise volume between the two groups. If the present exercise intervention was to counteract cancer-related fatigue and maintain physical fitness and physical activity levels more completely also during chemotherapy, better adherence to the program is probably necessary. Adherence is a critical component to the success of an exercise program and has been identified as a challenge in exercise intervention research, influencing treatment outcome and effectiveness [33, 34]. Low adherence rates might also reflect deficiency of the exercise prescription applied in this study [35] and the exercise environment with lack of supervision and behavioral change techniques [36–38]. Exercise adherence could have been increased by greater attention to the principles of building progression in the exercise program [35], and to apply individual goal setting based on the patient's reaction to chemotherapy. However, previous research evidence concerning the exercise dose sufficient to maintain physical activity and effectively counteract fatigue in all phases of breast cancer treatment is sparse and calls for more research.

Of note, an accrual rate of 72% of eligible patients is a strength of this study and is slightly higher compared to previous exercise research studies among cancer populations range, <40–57% [39]. High accrual rates increase the representativeness of the sample, ensuring external validity [40]. Also, an acceptable drop-out rate within the range (9–21%) reported in exercise interventions to breast cancer populations was obtained [38]. The issue on drop-outs in randomized control trials is especially a challenge for statistical conclusion validity. The sample was homogeneous at baseline and at Post_2_, which indicates that results are not affected by selection bias [41].

## 5. Conclusion

In summary, our hypothesis of additional effects of a home-based moderate-intensity exercise intervention performed by breast cancer patients during adjuvant chemotherapy treatment was not supported. Instead, the findings suggest that generally recommended physical activity levels of 150 minutes/week of moderate to vigorous physical activity [1] is enough to sufficiently relieve cancer-related fatigue and restore physical fitness and activity levels. Although it should be taken into consideration that the fatigue experience might have been reduced due to passing of time since chemotherapy, clinicians should routinely communicate to patients that being physicaly active on a regular basis can be beneficial to improve health and well-being. The results of this study can be used to guide nursing professionals to inform and motivate women with breast cancer to initiate and maintain exercise as a health behavior during chemotherapy according to guidelines provided for breast cancer populations.

## Figures and Tables

**Table 1:** Demographics and characteristics of study population (*N* = 67).

Variable	Total sample (*N* = 67)	Intervention group (*N* = 33)	Control group (*N* = 34)	*P* value
	*n* (%)	*n* (%)	*n* (%)	
Age (years)				0.576
Mean ± SD	52.2 ± 9.3	50.8 ± 9.7	53.6 ± 8.8	
Body weight (kg)				0.178
Mean ± SD	70.5 ± 13.8	69.0 ± 11.6	72.0 ± 15.7	
Waist line (cm)				0.444
Mean ± SD	83.1 ± 11.1	81.6 ± 9.9	84.5 ± 12.0	
Living conditions				
Living alone	10 (14.9)	5 (15.2)	5 (14.7)	0.510
Living with partner	54 (80.6)	27 (81.8)	27 (79.4)	0.204
Living with others	2 (3.0)	0 (0)	2 (5.9)	0.086
Missing	1 (1.5)	1 (3.0)	0 (0)	
Ethnicity				0.638
Norwegian	57 (85.1)	27 (81.8)	30 (88.2)	
Other	9 (13.4)	5 (15.6)	4 (11.8)	
Missing	1 (1.5)	1 (3.0)	0 (0)	
Children living at home				0.205
Yes	29 (43.3)	17 (53.1)	12 (35.3)	
No	37 (55.2)	15 (46.9)	22 (65.7)	
Missing	1 (1.5)	3.0 (1)	0 (0)	
Education				0.186
High school	12 (17.9)	4 (12.5)	8 (23.5)	
College	23 (34.3)	9 (28.2)	14 (41.1)	
University	31 (46.3)	19 (59.3)	12 (35.2)	
Missing	1 (1.5)	1 (3.0)	0.0 (0)	
Currently employed				0.331
Yes	19 (28.4)	12 (40.0)	7 (21.2)	
Yes, part time	14 (20.9)	5 (16.7)	9 (27.3)	
No	30 (44.8)	13 (43.3)	17 (51.5)	
Missing	4 (6.0)	3 (9.03)	1 (2.9)	
Cancer stage^a^				0.394
I	19 (31.7)	7 (24.2)	12 (38.7)	
II	34 (56.7)	19 (65.5)	15 (48.4)	
III	7 (11.6)	3 (10.3)	4 (12.9)	
PgR status				0.782
Negative	32 (47.8)	16 (48.5)	16 (47.1)	
Positive	35 (52.2)	17 (51.5)	18 (52.9)	
ER status				0.464
Negative	21 (31.3)	13 (39.4)	8 (23.5)	
Positive	46 (68.7)	20 (60.6)	26 (76.5)	
HER-2 status				0.254
Negative	55 (82.0)	26 (78.8)	29 (85.3)	
Positive	11 (16.4)	7 (21.2)	4 (11.8)	
Missing	1 (1.5)	0.0 (0)	1 (2.9)	
Surgery				0.866
Lumpectomy	22 (32.8)	21 (63.6)	24 (70.6)	
Mastectomy	45 (67.2)	12 (36.4)	10 (29.4)	
Chemotherapy regimen				0.898
FEC-60	33 (49.3)	16 (48.5)	17 (50.0)	
FEC-100	4 (6.0)	2 (6.1)	2 (5.9)	
FEC-60 + Taxotere	18 (26.9)	8 (24.2)	10 (29.4)	
FEC-100 + Taxotere	5 (7.5)	3 (9.1)	2 (5.9)	
FEC-60 + Taxol	7 (10.4)	4 (12.1)	3 (8.8)	
Other adjuvant systemic treatment	56 (93.3)	27 (93.1)	29 (93.5)	0.612
Radiotherapy	48 (71.7)	22 (75.9)	26 (83.9)	
Intervention duration (weeks)				0.807
Mean ± SD	17.2 ± 7.7	16.7 ± 7.6	17.6 ± 7.9	

SD: standard deviation, ER: estrogen receptor, PgR: progesterone receptor, FEC-60: chemotherapy regimen of fluorouracil, epirubicin and cyclophosphamide administered in 60 mg/m^2^ dosage. FEC-100: chemotherapy regimen of fluorouracil, epirubicin and cyclophosphamide administered in 100 mg/m^2^ dosage, HER-2: human epidermal growth factor receptor 2.

^a^Cancer stage based on pTNM staging system.

**Table 2:** Mean scores and standard deviations for study variables at baseline, end of chemotherapys and 6-month follow-up tests for the intervention and control groups.

	Intervention group			Control group		
	Baseline (*n* = 33)	End of chemotherapy Post_1_ (*n* = 29)	Followup Post_2_ (*n* = 25)	Baseline (*n* = 34)	End of chemotherapy Post_1_ (*n* = 31)	Followup Post_2_ (*n* = 28)
Schwartz Cancer Fatigue Scale-6^a^	10.28 (3.93)	12.01 (4.38)	10.43 (3.27)	11.36 (3.56)	13.13 (4.47)	10.42 (3.21)
6-Minute Walk Test^b^	656.89 (63.30)	644.02 (63.30)	678.62 (73.27)	638.64 (57.44)	628.33 (60.44)	643.39 (54.00)
MET-minutes/week^c^	1333.66 (1367.67)	1621.12 (1734.42)	2105.63 (2104.75)	1138.00 (1148.81)	1018.97 (1396.25)	1844.94 (1555.35)

^a^Fatigue scores ranging from 6 to 30.

^b^Reported in meters.

^c^Self-reported physical activity level (International Physical Activity Questionnaire short form) measured in metabolic equivalent of task (MET) minutes.

**Table 3:** Tests of baseline–end of chemotherapy (Post_1_) changes.

	Time (baseline–Post_1_)			Time × condition		
	df	*F* value	*P* value	df	*F* value	*P* value
Schwartz Cancer Fatigue Scale-6^a^	1/58	9.604	0.003*	1/58	0.001	0.970
6-Minute Walk Test^b^	1/57	3.005	0.088	1/57	0.036	0.849
MET-minutes/week^c^	1/56	0.125	0.725	1/56	0.727	0.398

*<0.05.

^a^Fatigue scores ranging from 6–30.

^b^Reported in meters.

^c^Self-reported physical activity level (International Physical Activity Questionnaire-short form) measured in metabolic equivalent of task (MET) minutes.

**Table 4:** Tests of baseline–follow-up (Post_2_) changes.

	Time (Baseline–Post_2_)			Time × condition		
	df	*F* value	*P* value	df	*F* value	*P* value
Schwartz Cancer Fatigue Scale-6^a^	1/50	1.512	0.181	1/50	0.398	0.463
6-Minute Walk Test^b^	1/49	6.957	0.009*	1/49	1.383	0.245
MET-minutes/week^c^	1/48	14.77	0.000*	1/48	0.105	0.747

*<0.05.

^a^Score range 6–30.

^b^Reported in meters.

^c^Self-reported physical activity level (International Physical Activity Questionnaire-short form) measured in metabolic equivalent of task (MET) minutes.
